# Bleeders

**Published:** 1875-09

**Authors:** 


### "BLEEDERS."

The Boston *Transcript* says that "a strange story comes from Hamilton, Mass., about the "Bleeders," as they are called, of that town, a family whose members almost invariably die of bleeding. The legend connected with it is that in Salem witchcraft times, a sea captain brought his wife and little girl to town, leaving them with a Spanish nurse, who was a quick-tempered woman, and, being annoyed by the peevishness of the child, deliberately bled her to death by opening a vein in her arm at intervals, threatening her meanwhile with instant death if she told. The mother, after the death of the child, found out the cause, and fell into a decline, cursing, with her latest breath, her child's murderer, and predicting the same death to all her male descendants. An elderly woman who lives in the town is quoted as saying that, to her knowledge, five sons have met their deaths by bleeding, one by bleeding at the nose, and others by wounds which appeared slight, but which no efforts of the physicians could close." This is simply a coincidence, if there is indeed any truth whatever in the story of an uttered "curse." The family are simply of the hemorrhagic diathesis—that is to say, having a tendency to bleed profusely upon any injury to the skin or membranes. Thousands of families, weakened by disease or dissipation in their ancestors, are subject to bleedings of this character, and with such persons even the extraction of a tooth may be attended with fatal results. The blood is thin in such cases, and does not readily form a clot to close a severed blood-vessel. So the mouth of a wounded artery or vein lies open, and unless the injury is so situated that a ligature can be applied, the life ebbs away. But no "curses" uttered by the latest breath or any other breath, of a raving woman or any other person, has anything whatever to do with the case.

---

To Restore the Color of Carpets.—A table-spoonful of ammonia in one gallon of warm water will often restore the color of carpets, even if produced by acid or alkali. If a ceiling has been whitewashed with the carpet down, and a few drops should fall, this will remove it. Or, after the carpet is well beaten and brushed, scour with ox-gall, which will not only extract grease but freshen the colors. One pint of gall in three gallons of warm water will do a large carpet. Table and floor oil-cloths may be thus washed. The suds left from a wash, when ammonia is used, even if almost cold, cleans these new floor-cloths well.

---

The sick squaw of a dusky chief in Washington Territory lately told her noble husband that she didn't think that she should ever feel any better unless he killed her doctor. This is a novel and startling view of medical matters, and interesting to the profession. The doctor was duly killed; and upon being tried for his murder, the chief was acquitted on the ground that he acted in defense of his wife's life! The doctors in those regions must feel a little doubtful about continuing in the business under such circumstances.

